# Digital Information Technology Use and Transnational Healthcare: A Population-Based Study on Older Russian-Speaking Migrants in Finland

**DOI:** 10.1007/s10903-021-01301-9

**Published:** 2021-11-05

**Authors:** Young-Kyu Shin, Veera Koskinen, Anne Kouvonen, Teemu Kemppainen, Antero Olakivi, Sirpa Wrede, Laura Kemppainen

**Affiliations:** 1 Faculty of Social Sciences, University of Helsinki, Unioninkatu 37, PO Box 54, 00014 Helsinki, Finland; 2 Centre for Public Health, Queen’s University, Belfast, UK; 3 Department of Geosciences and Geography, University of Helsinki, Yliopistonkatu 3, PO Box 4, 00014 Helsinki, Finland; 4 Centre Maurice Halbwachs (CNRS/EHESS/ENS), Paris, France; 5 Swedish School of Social Science, University of Helsinki, Helsinki, Finland

**Keywords:** Transnational healthcare, Digital information technology, Migration, Migrants, Aging, Older adults, Integration, Discrimination

## Abstract

This study examines the association between digital information technology (DIT) use and the utilization of transnational healthcare (THC) in older migrants, and investigates how this relationship depends on social integration or perceived discrimination in health services in the destination country. The data from a population-based study conducted in Finland in 2019, which targeted Russian-speaking residents aged 50 and above (n = 1082) nationwide, are analyzed. The analysis demonstrates that those who had a high level of DIT use were significantly more likely to use THC than those who had a low level of use. However, the findings do not show that the relationship depends on social integration or perceived discrimination. Older migrants can actively use transnational networks to address their health and well-being issues by using DIT and seeking healthcare abroad. Their health service use can be illustrated as an active process involving various geographical domains.

## Background

Aging in a foreign land or ‘out of place’ is becoming more common due to population aging and increasing global migration [1]. The number of older foreign-born residents, as well as the ‘future older migrants’ (age group 45–54) is growing throughout Europe [2]. This development has fueled concerns related to the health of older migrants, who may face several barriers of access to formal and professional healthcare in their country of residence.

In this study, we study transnational healthcare (THC) from the perspective of older migrants drawing on a survey of Russian-speaking migrants aged 50 and above who were born in the Former Soviet Union and who currently live in Finland. We define THC as a wide array of practices in which healthcare service users and providers, as well as pharmaceuticals, health-related information, and knowledge, move across borders [3, 4]. Transnational healthcare can be seen as part of a broader phenomenon of people’s increasingly mobile lifestyles and the globalization of healthcare [5, 6]. Transnational networks are an important source of information and assistance for many older migrants [7]. The multiple ways in which migrants combine and use health-related knowledge and services in the transnational context has only recently gained scholarly attention [8–11]. Transnational healthcare may be an option that helps migrants find preferred forms of care. However, some migrants may find THC as their only option due to barriers of access to healthcare, such as inadequate language skills, insufficient knowledge of the healthcare system, or fear of discrimination [8, 10, 12].

Recent studies have found that migrants seek health services and health-related information within transnational social networks that are viewed via digital information technologies (DIT), such as smartphone applications, email, and the internet in general [13]. Digital information technologies are important for the overall well-being of older migrants, especially if their close relatives live in another country [11, 14]. Importantly, DIT facilitates an information search on available healthcare service options as well as their evaluation (e.g., quality, costs, and access) and the comparison between different services [15]. However, although older adults’ use of DIT varies greatly, aging may weaken the ability to use and adopt new DIT and thereby narrow the healthcare options available for people excluded from it [16].

Russian speakers are the largest foreign language group in Finland, comprising almost 21% (around 79,000 persons) of all foreign language speakers [17]. The Russian speakers—those who have migrated to Finland from several different countries that belonged to the former Soviet Union—actively take part in transnational practices and tend to socially be better integrated into the Finnish society compared to many other foreign-born minority groups in Finland [18]. This is usually explained by both geographical and cultural proximity [19]. In addition, Finland was an autonomous part of the Russian Empire from 1809 to 1917. Throughout history, there has been trade, cooperation, and everyday encounters between the citizens of Finland and Russia, especially at the border regions. As it is relatively easy and inexpensive to travel from Finland to Russia, the use of healthcare services in Russia has become common among the working-age Russian-speaking migrants in Finland for the last decade [10]. However, Russia may not be the only option for THC among Russian speakers, as they may look for health services, for example, in the Baltics or Eastern Europe, where there are also sizable Russian-speaking populations.

Against this background, we analyze recent population-based survey data on older Russian-speaking migrants in Finland to examine the prevalence and determinants of THC with a special focus on DIT. We fill the following gaps in prior research: first, the implications of DIT for THC has not received prior attention in any population; second, there is a scarcity of research on older migrants’ use of THC, and high-quality qualitative evidence based on representative data is very rare in THC literature. Furthermore, we address the roles of perceived discrimination, such as unequal treatment and disrespect in health services and social integration in the destination country regarding the use of THC, which also remain understudied topics.

## Conceptual Framework

Transnational healthcare is particularly common among migrant populations [8, 9, 19]. According to prior studies, practical issues such as geographical proximity, cultural familiarity, and lower prices motivate migrants to utilize health services in their country of origin [11, 20, 21]. In addition, weak local language skills, poor health status, as well as discrimination and experienced inconveniences (e.g., cross-cultural misunderstanding and differing expectations of care) experienced in the healthcare system of the country of residence may encourage people to return ‘home’ for care [8, 9, 20, 22]. Health services of the origin and destination countries are often used in parallel and not necessarily as mutually exclusive alternatives [10, 12].

Using a survey from 2011 on working-age Russian-speaking migrants in Finland, Kemppainen et al. [10] illustrated that experiencing discrimination in healthcare services in the destination country is associated with the use of THC. The study also found that low social integration in the destination country encouraged the utilization of THC. Kemppainen et al. [18] showed that, among working-age migrants, the older age groups (55–64 years old) are less integrated into their destination societies than the younger age groups. Older migrants are exposed to specific conditions and risks of exclusion and social isolation [1]. They are also likely to face multiple forms of marginalization in healthcare services in their destination countries [14].

In addition to exploring how DIT is related to THC, our data allows for examining the relationship of THC with integration and discrimination experienced in health services among older migrants, which remains an understudied question. Moreover, we are able to analyze whether integration or discrimination modify the association of DIT and THC: those who are more strongly integrated to the destination society may use DIT to access local health services, whereby the association between DIT and THC should be weaker. In other words, the socially less integrated, older migrants could use DIT specially to access THC. Moreover, they may need to learn to use DIT to access THC. With a similar rationale, the experience of discrimination in the health services of the destination country may strengthen the association between DIT and THC.

## Methods

### The Data

This article analyzes the data collected by the Care, Health and Ageing of Russian-speaking Minority in Finland (CHARM) study which focuses on Russian-speaking migrants who are 50 years of age or older and permanently residing in Finland. The data were collected in May and June, 2019. A random sample of 3000 people was drawn from the Population Register. The sample was stratified by gender. Of those invited, 36% responded to the survey and thus the final sample size was 1082. The Finnish Tax Administration register data from 2017 were used to model the non-response rate and to compute the weights [23].

### Variables and Statistical Models

The dependent variable stems from the item that asks whether the participant received medical treatment or help in countries other than Finland during the last 12 months (0 = no, 1 = yes).

A multiple correspondence analysis (MCA) was employed as a dimension reduction method to create the variables indicating (1) the use of DIT, and (2) social integration in the destination country. The MCA is considered the counterpart of factor analysis and principal component analysis for categorical variables [24]. We ran the MCA using Burt’s approach and extracted standard normal coordinates [24, 25].

To create the indicator of the participants’ use of DIT, we first conducted a MCA based on 17 items related to the participants’ use of DIT in various situations. Although the first dimension of the MCA solution explained about 92% of the total variance, we did not directly use it as the variable for the use of DIT as the items for this MCA had a number of missing values. Instead, we created a categorical variable for the indicator by including the category of non-response to provide more power for the other independent variables. As a result, the indicator of the use of DIT consists of three values (low level of use, high level of use, and non-response). Specifically, a case is treated as low level of DIT use if its predicted row score for the first dimension is below zero (i.e., the mean of the MCA indicator score), while it is regarded as high level of DIT use if its score is zero or larger.

The degree of social integration in Finland is also measured based on a MCA of the following categorical variables: Finnish citizenship, the length of residence in Finland, education in Finland, the frequency of following Finnish media, and the command of Finnish or Swedish languages. The first dimension of the MCA result explains about 82% of the total variance in those items.

The binary variable indicating the experience of discrimination is created by recoding an ordinal variable inquiring how often the participant experienced worse service than others by a doctor or in a Finnish health center or hospital (0 = never, 1 = rarely, fairly often, or very often).

The control variables in the model were the following: gender (0 = man, 1 = woman), age (0 = under age 65, 1 = aged 65 or above), education obtained in the home country (0 = non-tertiary education, 1 = tertiary education), marital status (0 = divorced or living separately, widow or widower, or not married, 1 = married or cohabitating), employment status (0 = not in paid work, 1 = in paid work), monthly household net income (0 = less than €2499, 1 = €2500 or higher), financial hardship (0 = No, 1 = Yes), chronic illness (0 = none, 1 = having a chronic disease, such as high blood pressure, diabetes, or asthma), the use of Finnish healthcare services during the past 12 months (0 = non-use, 1 = use), and visiting the country of origin during the past 12 months (0 = no visit, 1 = visit).

To investigate the relationships of social integration in Finland, experienced discrimination, and the use of DIT with accessing THC, logistic regression models including these independent and control variables were first estimated. After that, models adding the interaction terms between the destination-country social integration and DIT and between discrimination and DIT were separately estimated to examine these interaction effects. In the models including interaction terms, cases corresponding to no response in DIT use variable are excluded in order to focus on the marginal effects of DIT use in connection with destination-country social integration and experienced discrimination. Adjusted sampling weights and stratification of the design are applied to statistical models.

## Results

Table 1 displays the descriptive results. A quarter of the participants used healthcare services outside Finland during the last 12 months. The main explanatory variable, DIT use, is a categorical one which consists of three groups: low level of use (23%), high level of use (36%), and non-respondents (41%). When it comes to the items used to create the destination-country social integration indicator, nearly half (49%) of the participants have a Finnish citizenship, while only 37% have received education in Finland. More than a half have resided in Finland for over 10 years, and around 60% have a poor command of the Finnish or Swedish languages. In addition, over 70% of the respondents keep up with the Finnish media at least once a week. Regarding the variable of experienced discrimination, 17% of the participants report that they have experienced discrimination related to healthcare services in Finland.

**Table 1:** Descriptive statistics

	%	Total number of respondents
*Outcome*		
**Accessing healthcare abroad (outside Finland)**	25.1	1053
*Explanatory variables*		
**Digital information technology (DIT) use**		1082
Low	23.1	
High	36.2	
No response	40.7	
Predicted score from the dimension for DIT use based on MCA (mean)	0.000	
Predicted score from the dimension for DIT use based on MCA (SD)	1.001	
1. Smartphone	84.6	965
2. Tablet PC	54.5	841
3. Laptop or PC	85.7	982
4. Electronic identification	78.4	937
5. Frequency of Internet use		1067
Never use	6.8	
Less than once a week	2.6	
At least once a week	3.4	
(Almost) Every day	29.8	
Several times a day	57.4	
*Reasons for internet use*		
6. Messages or calls	87.2	981
7. Banking	87.0	1010
8. Reading news	90.7	1033
9. Social media	63.1	1000
10. Accessing health and social care	57.6	975
11. Accessing other public services	58.7	963
12. Viewing personal health data	37.2	948
13. Looking for health information	66.3	980
14. Accessing healthcare services and consultations from abroad	11.6	933
15. Contact with peer support groups	19.1	943
16. Comparing healthcare services (quality and prices)	24.9	943
17. Other	67.3	906
**Destination-country social integration**		
Predicted score from the dimension for social integration in Finland based on MCA (mean)	0.000	906
Predicted score from the dimension for social integration in Finland based on MCA (SD)	1.001	
1. Finnish citizenship	49.0	1071
2. Received education in Finland	37.4	1069
3. The length of residence in Finland		1052
< 5 years	25.5	
5–9 years	21.4	
10–14 years	21.0	
15 + years	32.1	
4. Following Finnish media		998
Daily	45.4	
Weekly	26.8	
Monthly	9.3	
Less often than monthly or never	18.4	
5. Command of Finnish or Swedish language		1019
Never use	11.5	
Basic	50.7	
Medium	13.9	
High	23.9	
**Discrimination**		
Experience of discrimination in Finnish healthcare services	16.7	1043
*Control variables*		
Woman	43.1	1082
Age: 65 years or older	39.7	1082
Tertiary education	50.6	1082
Married or cohabiting	73.6	1082
In paid work	39.4	1082
High monthly household income: more than €2499	19.7	1082
Financial hardship	34.5	1064
Chronic illness	82.8	1082
Accessed healthcare services in Finland during the last 12 months	84.6	1082
Visited country of origin during the last 12 months	79.1	1075

Table 2 shows the results of the logistic regression analyses of the use of THC. The first and second columns in the table report the outcomes drawn from bivariate models and the full model, respectively. The findings reveal that all key explanatory variables are associated with the use of THC in both bivariate and full models. As expected, DIT use is positively associated with THC: those with a high level of DIT use are significantly more likely to use THC than those who have a low level of DIT use. Having a high level of DIT use increases the odds of using THC by 62% in the bivariate model and 86% in the full model.

**Table 2:** Estimates of logistic regression models on accessing THC

	I: Bivariate models			II: Full model		
	OR	Sig.	95% CI	OR	Sig.	95% CI
Degree of DIT use						
Low	Ref.			Ref.		
High	1.62	*	1.05 − 2.52	1.86	*	1.10 − 3.17
No response	1.05		0.68 − 1.63	1.54		0.90 − 2.62
Destination-country social integration (MCA)	0.78	**	0.65 − 0.94	0.68	***	0.55 − 0.84
Discrimination	1.81	**	1.22 − 2.69	1.86	**	1.16 − 3.07
Woman	1.74	***	1.30 − 2.33	2.13	***	1.48 − 3.07
Age: 65 years or older	0.82		0.59 − 1.14	0.78		0.50 − 1.22
Tertiary education	1.40	*	1.01 − 1.94	1.55	*	1.04 − 2.30
Married or cohabiting	1.30		0.91 − 1.86	1.26		0.80 − 1.99
In paid work	0.81		0.58 − 1.12	0.68		0.41 − 1.12
High monthly household income: More than €2499	1.11		0.74 − 1.67	1.58	*	0.91 − 2.76
Financial hardship	0.79		0.56 − 1.12	0.94		0.61 − 1.44
Chronic illness	2.84	***	1.69 − 4.78	3.11	***	1.69 − 5.72
Accessed healthcare services in Finland during last 12 months	0.87		0.54 − 1.39	0.66		0.38 − 1.15
Visited origin country during last 12 months	7.90	***	4.10 − 15.22	5.54	***	2.75 − 11.14
Intercept				0.01	***	0.00 − 0.04
*N*				826		

*OR* odds ratio; *Sig*. significance; *CI* confidence interval; *Ref*. reference category

***p < 0.001, **p < 0.01, *p < 0.05

Moreover, a one-unit increase in the destination-country social integration indicator decreases the odds of accessing THC by 22% in the bivariate and 32% in the full model. As a result, it can be concluded that the more socially integrated a person is in Finnish society, the less likely they are to travel abroad for medical purposes. Finally, participants who experienced discrimination in Finnish healthcare services are significantly more likely to use THC than those without such a negative experience. The odds ratios are 1.81 and 1.86 in the binary and full models, respectively.

The estimates of the models with their interaction terms are displayed in Table 3. The interaction terms between DIT use and destination-country social integration (model A) and between DIT use and experienced discrimination (model B) were not statistically significant. Moreover, the average marginal effects of DIT use do not significantly change by either the degree of social integration in Finland or by the experience of discrimination (see Appendices 3 and 4). Consequently, the findings did not provide any evidence that the relationship between DIT use and accessing THC varies depending on destination-country social integration or experienced discrimination.

**Table 3:** Estimates of logistic regression models with interactions terms on accessing THC

	Model A			Model B		
	Coefficient	Sig.	Standard error	Coefficient	Sig.	Standard error
Degree of DIT use						
Low	Ref.			Ref.		
High	0.899	**	(0.331)	0.809	*	(0.325)
Destination-country social integration (MCA)	− 0.826	**	(0.267)	− 0.615	***	(0.140)
**Destination-country social integration × DIT use**						
Social integration in Finland × High use	0.279		(0.295)			
Discrimination	0.491		(0.341)	0.401		(0.580)
**Discrimination × DIT use**						
Discrimination × High use				0.090		(0.677)
*N*	553			553		

Estimates of control variables are not displayed, as they did not show a significant change from those in the full model without interactions terms

*Sig*. significance; *Ref*.  reference category

***p < 0.001, **p < 0.01, *p < 0.05

## Discussion

This study examines the THC use of older Russian-speaking migrants in Finland in terms of their DIT use, social integration in Finland, and discrimination experience, which fills several gaps in prior literature. We find that 25% of the participants used healthcare services abroad (outside Finland) during the last 12 months. Women accessed THC more often than men. Tertiary education, having a high household income, and suffering from chronic illness also increase the likelihood of accessing THC. However, age, marital status, employment status, and economic hardship do not make difference. In addition, the results show that Russian-speaking migrants in Finland over the age of 50 are actively using the Internet, especially for health information and healthcare seeking purposes.

As expected, the findings demonstrate that the level of DIT use is positively associated with accessing THC. Contrary to our expectations, social integration and discrimination do not modify this association. However, weak social integration and experienced discrimination in Finnish healthcare services are, as such, positively related to THC. Thus, our study lends support to previous findings indicating close ties with migrants’ THC use and their integration experience in the destination country [10, 21].

Earlier studies examined older migrants as recipients of care in transnational families and networks [14]. Our study challenges and complements this approach by emphasizing the agency side of the phenomenon and asking how older adults with migration backgrounds also actively resolve issues related to their own health and well-being, for example, by using DIT and seeking healthcare abroad. This way, we depict the health service use of older migrants as an active process involving various geographical domains [18], and a complex interaction of virtual and real transnational spaces. Our results suggest that, although older adults with migrant backgrounds would be more likely to feel challenged in accessing THC compared to younger people, it is probable that they can overcome this challenge if they use DIT for multiple purposes. As outlined in previous research, transnational networks are a significant source of well-being, particularly for older migrants, and DIT contributes a great deal in maintaining and acting within these networks [4, 26]. Thus, it may be that the need and desire to maintain these networks also motivates the adoption of new forms of DIT and the use of them for multiple purposes.

From the industrial perspective, the advancement of DIT in healthcare seems to act as a facilitator to help migrants access THC and could extend the business field by promoting it. In addition, it can contribute to the improvement of customer utility and satisfaction by providing migrants with further options. However, it may also hinder their integration into the destination country, and cause disconnection with their local healthcare services. As a preferred choice, THC can contribute to one’s sense of agency. However, if one is forced to search for medical help from abroad due to discrimination or lack of understanding of the local healthcare system, the services can be seen as non-inclusive. This situation requires the healthcare system of the residence country to make a more deliberate effort to provide equal access and appropriate care by considering people’s diverse cultural backgrounds [10].

We did not find that the interaction terms between DIT use and the other explanatory variables are significant. Older Russian-speaking migrants in Finland use DIT to access THC, regardless of their social integration in Finnish society or their discrimination experiences. Thus, the combination of push factors (weak integration and discrimination) with good DIT capacities does not make an additional impact on the likelihood of using THC. However, future research should analyze such interactions, as this study focuses only on older adults and analyzes the data with a high rate of non-responses in the DIT use variable.

This study provides a novel insight into the dynamics of THC in terms of population aging and DIT advancement. Given the findings, we can expect that, as DITs have gradually advanced and become easy to use, older adults with migration backgrounds are likely to more frequently and actively access THC by employing these technologies, unless healthcare systems in their destination countries are improved to also meet their needs.

## Contribution to the Literature

While previous studies have mainly examined older migrants as recipients of care in transnational families and networks, this study challenges and complements this approach by exploring how older adults with migration backgrounds actively resolve issues related to their own health and well-being. Furthermore, this work contributes to evidence of the association between the use of DIT and access to THC among migrants in the current situation where digitalization has become more dominant in healthcare services. Our findings carry significant implications concerning how to design and improve digitalized healthcare services for migrants. In organizing healthcare services, equal access and culturally competent care should be guaranteed and services should be available for those with lower or no skills in DIT.

## Appendix 1

MCA biplots of variables for DIT use.

## Appendix 2

MCA biplots of variables for social integration.

## Appendix 3

Average marginal effects of DIT use by social integration.

## Appendix 4

Average marginal effects of DIT use by experienced discrimination.
